# A Phage Display Selected 7-mer Peptide Inhibitor of the *Tannerella forsythia* Metalloprotease-Like Enzyme Karilysin can be Truncated to Ser-Trp-Phe-Pro

**DOI:** 10.1371/journal.pone.0048537

**Published:** 2012-10-31

**Authors:** Peter Durand Skottrup, Grete Sørensen, Miroslaw Ksiazek, Jan Potempa, Erik Riise

**Affiliations:** 1 Biomolecular Interaction Group, Department of Drug Design and Pharmacology, Faculty of Health and Medical Sciences, University of Copenhagen, Copenhagen, Denmark; 2 Department of Microbiology, Faculty of Biochemistry, Biophysics and Biotechnology, Jagiellonian University, Krakow, Poland; 3 Oral Health and Systemic Diseases Research Group, University of Louisville, School of Dentistry, Louisville, Kentucky, United States of America; University of Houston, United States of America

## Abstract

*Tannerella forsythia* is a gram-negative bacteria, which is strongly associated with the development of periodontal disease. Karilysin is a newly identified metalloprotease-like enzyme, that is secreted from *T. forsythia*. Karilysin modulates the host immune response and is therefore considered a likely drug target. In this study peptides were selected towards the catalytic domain from Karilysin (Kly18) by phage display. The peptides were linear with low micromolar binding affinities. The two best binders (peptide14 and peptide15), shared the consensus sequence XWFPXXXGGG. A peptide15 fusion with Maltose Binding protein (MBP) was produced with peptide15 fused to the N-terminus of MBP. The peptide15-MBP was expressed in *E. coli* and the purified fusion-protein was used to verify Kly18 specific binding. Chemically synthesised peptide15 (SWFPLRSGGG) could inhibit the enzymatic activity of both Kly18 and intact Karilysin (Kly48). Furthermore, peptide15 could slow down the autoprocessing of intact Kly48 to Kly18. The WFP motif was important for inhibition and a truncation study further demonstrated that the N-terminal serine was also essential for Kly18 inhibition. The SWFP peptide had a Ki value in the low micromolar range, which was similar to the intact peptide15. In conclusion SWFP is the first reported inhibitor of Karilysin and can be used as a valuable tool in structure-function studies of Karilysin.

## Introduction

Periodontitis is a serious bacterial infection-driven inflammatory disease affecting the periodontium, i.e., the tissues that surround and support the teeth. The ‘red complex’ is a term used for the three bacterial taxa that are considered the major periodontopathogens (*Treponema denticola*, *Porphyromonas gingivalis* and *Tannerella forsythia*) that lead to disease development [1]. These pathogens all produce high levels of extracellular proteolytic activity [2]–[8], which contributes to the periodontitis symptoms; loss of attachment between the tooth and the gingiva, which is due to bone degradation and weakening of the soft tissues surrounding the root of a tooth. This ultimately leads to formation of deep periodontal pockets and teeth loss [9]. Furthermore, accumulating evidence suggests that severe forms of periodontitis contribute to development of the systemic diseases, stroke, diabetes and rheumatoid arthritis [10]–[14]. Current periodontitis therapies are based upon mechanical removal of supra- and subgingival bacterial plaque from the tooth surface in conjunction with the use of antibiotics. Unfortunately, this treatment is not always fully effective, and focus was therefore directed towards development of protease inhibitors as an alternative weapon towards the periodontopathogen effects. In fact, a factor Xa inhibitor could inhibit the *P. gingivalis* gingipain proteases RgpA and RgpB and was bactericidal towards *P. gingivalis* [15]. Furthermore, chlorhexidine and benzamidine were used successfully to inhibit *P. gingivalis* gingipains [16], [17]. Furthermore, doxycycline could inhibit protease activity from *P. gingivalis* and *T. denticola* [18].

*T. forsythia* is a gram-negative bacteria, which secrete proteases that act as virulence factors (PrtH, a cysteine protease and BspA, a trypsin-like protease). PrtH displays hemolysin activity and one study suggested that BspA mediates *T. forsythia* attachment to fibronectin and fibrinogen [19]–[21]. Karilysin, a newly identified metalloprotease isolated from *T. forsythia* [6] is secreted as a 472-residue protein and has the following composition; a 20-residue signal sequence, a 14-residue pro-peptide, an 18 kDa catalytic peptidase domain and a 30 kDa C-terminal domain of unknown function. The full length enzyme could be recombinantly expressed but the enzyme matured through sequential autolysis, by first generating a fully active 48 kDa variant, followed by formation of the catalytic domain (named Kly18) [6], [22]. Kly18 structural analysis demonstrated a similarity to mammalian matrix metalloproteinases (MMPs) [22]. MMPs are a separate family within the ‘metzincin’ clan of MPs and participate in turnover of extracellular-matrix components and selective activation/inactivation of other proteins and enzymes. The MMP activity is tightly regulated, however uncontrolled MMP-proteolysis can occur, leading to tissue destruction, apoptosis and inflammation [23]. Metalloproteases derived from microbial pathogens were documented as important virulence factors contributing to evasion of antimicrobial mechanisms of the innate immune system [24].

Only limited information is available on the biology of Karilysin. Recent data demonstrated that Karilysin can inactivate the antimicrobial peptide LL-37 by proteolytic cleavage [7]. This suggests that Karilysin can contribute to evasion of the human immune response. This hypothesis has been further substantiated by recent findings that Karilysin-expressing *T. forsythia* isolates inhibits all pathways of the complement system by Karilysin-mediated degradation of complement system proteins (mannose-binding lectin, ficolin-2, ficolin-3, C4 and C5) [25]. For these reasons Karilysin is considered a potential target for therapeutic intervention but no Karilysin inhibitors currently exist.

In this study phage display was used to identify a peptide that specifically bound Karilysin and efficiently inhibited the proteolytic activity of Karilysin.

**Figure 1:**
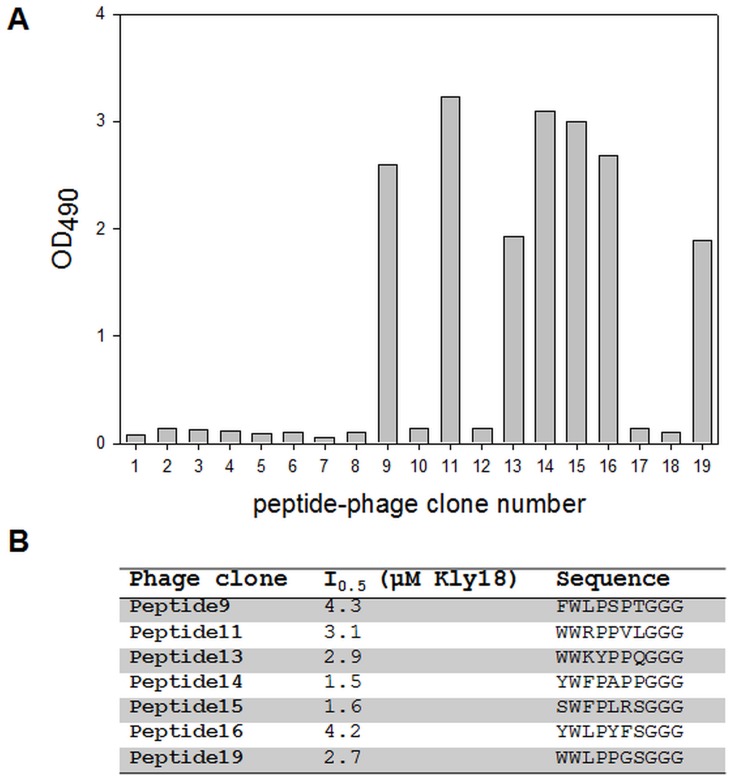
Phage ELISA test of nineteen selected clones after four panning rounds, peptide sequences and estimated apparent afffinity. A) Optical density responses detected after the binding of peptide-phage to immobilized Kly18 are shown. Background binding to BSA has been subtracted for each clone. Clones with signals above 0.5 were deemed positive and further sequenced. B) Peptide-phage clones were tested in inhibition ELISA for estimation of affinity. All clones (except clone 13) shared the WXP motif.

## Materials and Methods

### Miscellaneous Reagents

Karilysin catalytic domain (Kly18) and intact Kly48 were produced as previously described [6]. Active human MMP-3 catalytic domain, Bovine Serum Albumine (BSA), LB-medium and FITC-Casein were from Sigma-Aldrich. Maltose-binding protein (MBP) was from ProSpec-Tany TechnoGene Ltd. Peroxidase conjugated mouse anti-M13 phage monoclonal antibody, LMW (Low Molecular Weight)-SDS Marker and 1 ml MBPTrap HP columns were from GE-Healthcare. Peptide phage libraries (7-mer and 7-mer cysteine-constrained), pMAL-pIII vector, M13KE insert extension primer (NEB #E8101), −96 gIII sequencing primer (NEB #S1259), monoclonal anti-MBP HRP-conjugate, *EagI* and *Acc65I* were from New England Biolabs. Maxisorp microtiter plates and black fluorescence non-surface treated plates were both from NUNC. OPD-tablets (*o*-Phenylenediamine dihydrochloride) were from DAKO. All peptides were synthesised by Genscript. T4-DNA ligase was from Invitrogen. QIAquick gel extraction kit was from QIAgen. Ampicillin was from Calbiochem. Isopropyl β-D-thiogalactoside (IPTG) was from VWR.

**Figure 2:**
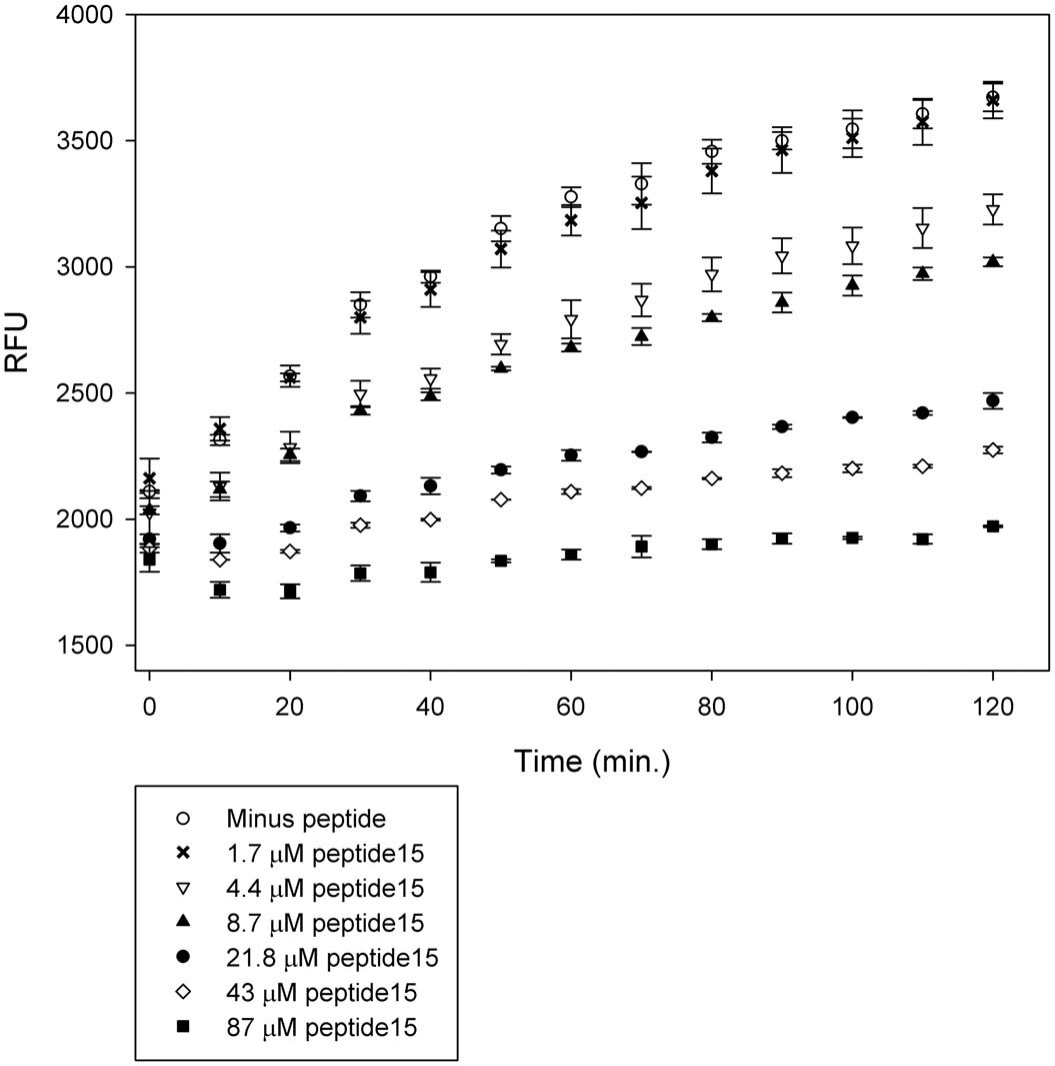
Peptide15 inhibits the proteolytic activity of Kly18. Peptide15 was pre-incubated with Kly18 in varying concentrations followed by addition of the substrate FITC-casein. The peptide15 inhibitory effect was seen as a decrease in Relative Fluorescence Units (RFU) in a dose-response manner. Error bars represent standard deviations between three experiments.

**Figure 3:**
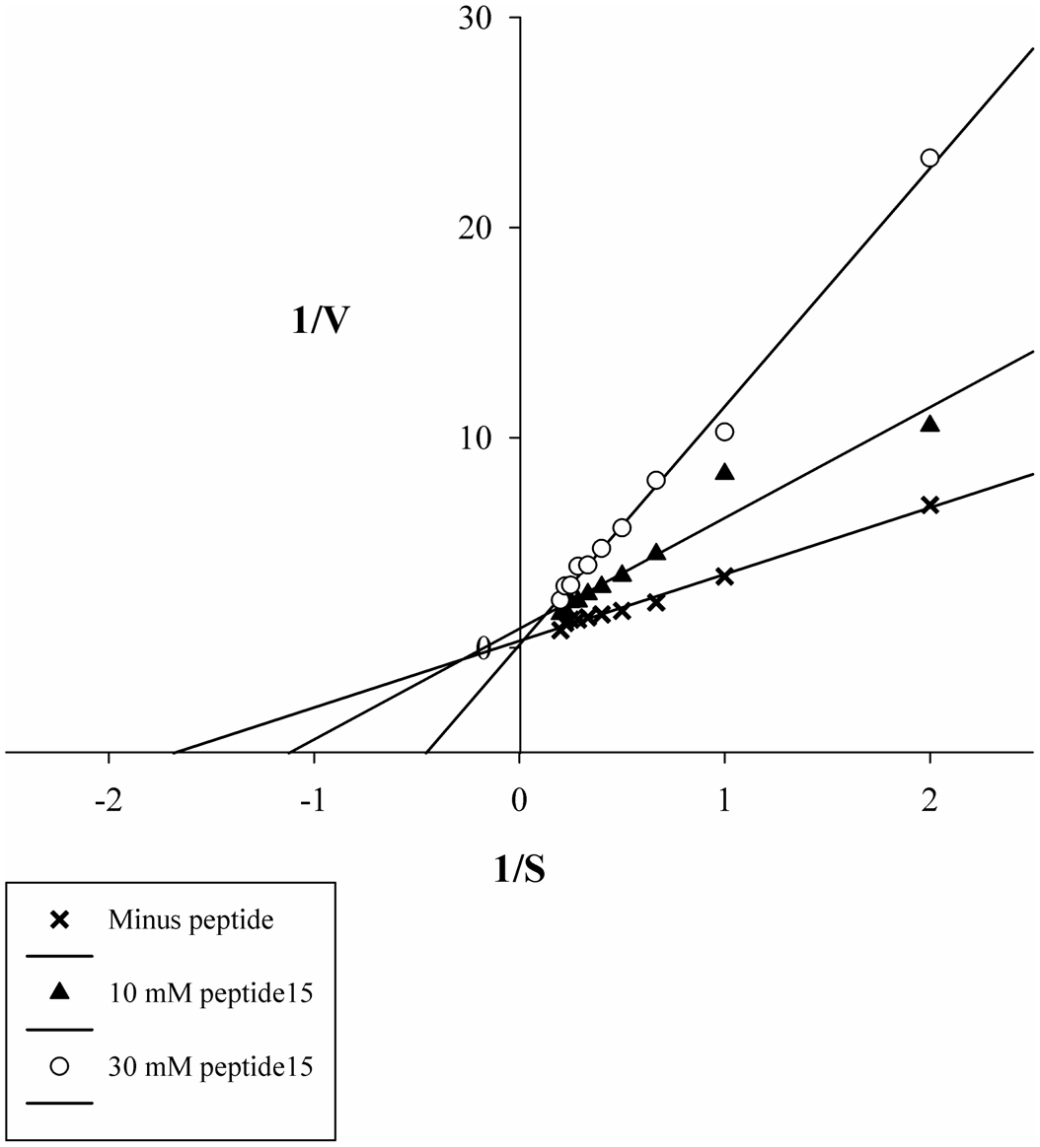
Peptide15 displays the characteristics of a competitive inhibitor. Shown are 1/V-1/S plot of peptide15 inhibition of Kly18. Kly18 was pre-incubated with fixed concentrations of peptide15 (10 µM and 30 µM) for 30 minutes, followed by incubation with varying concentrations of FITC-casein (5–50 µg/ml). As seen from the Lineweaver-Burke plot the Y-intercept is approximately the same for un-inhibited Kly18 as Kly18 inhibited with 10 µM and 30 µM, respectively. This suggested that peptide15 was a competitive inhibitor of Kly18. Linear regression lines were made using SigmaPlot 11.0. Data represent mean values from three experiments per substrate concentration.

### Biopanning

Phage libraries displaying seven amino acids in random sequence order at the N-terminal end of protein III were used for affinity selection of peptide binders towards Kly18. Both a linear and a constrained version of the peptide library were used for the selection. Microtiter plates were coated at 4°C for 16 hours with purified Kly18 at 0.66 µM using 100 µl per well. These plates were subsequently washed in PBS (20 mM sodium phosphate, 150 mM NaCl, pH 7.4) supplemented with 0.05% Tween-20 (PBS-T) and then blocked with 4% BSA in PBS for 2 h at room temperature. Bacteriophage at 10^11^ pfu/100 µl were used for each panning round. The constrained and linear libraries were mixed and panned together as a mixture in PBS supplemented with 0.1% Tween20, pH = 7.4. After incubation for one hour at room temperature plates were washed ten times with PBS-T (0.1% Tween20) and bound peptide-phage were eluted with glycine/HCl, pH = 2.2 for 10 min. followed by neutralisation with Tris–base, pH = 9.0. Eluted phage were used to infect exponentially growing TG1 cells overnight at 37°C. The following day peptide-phage were precipitated from the cell supernatant with phage precipitation buffer (20% (w/v) PEG6000), 2.5 M NaCl) and re-dissolved in PBS as described [26]. After four rounds of panning, single clones were isolated and tested for Kly18 binding in phage ELISA (see below). Single stranded DNA was extracted from positive clones according to [27] and the DNA was sequenced in the region corresponding to the random peptide region.

**Figure 4:**
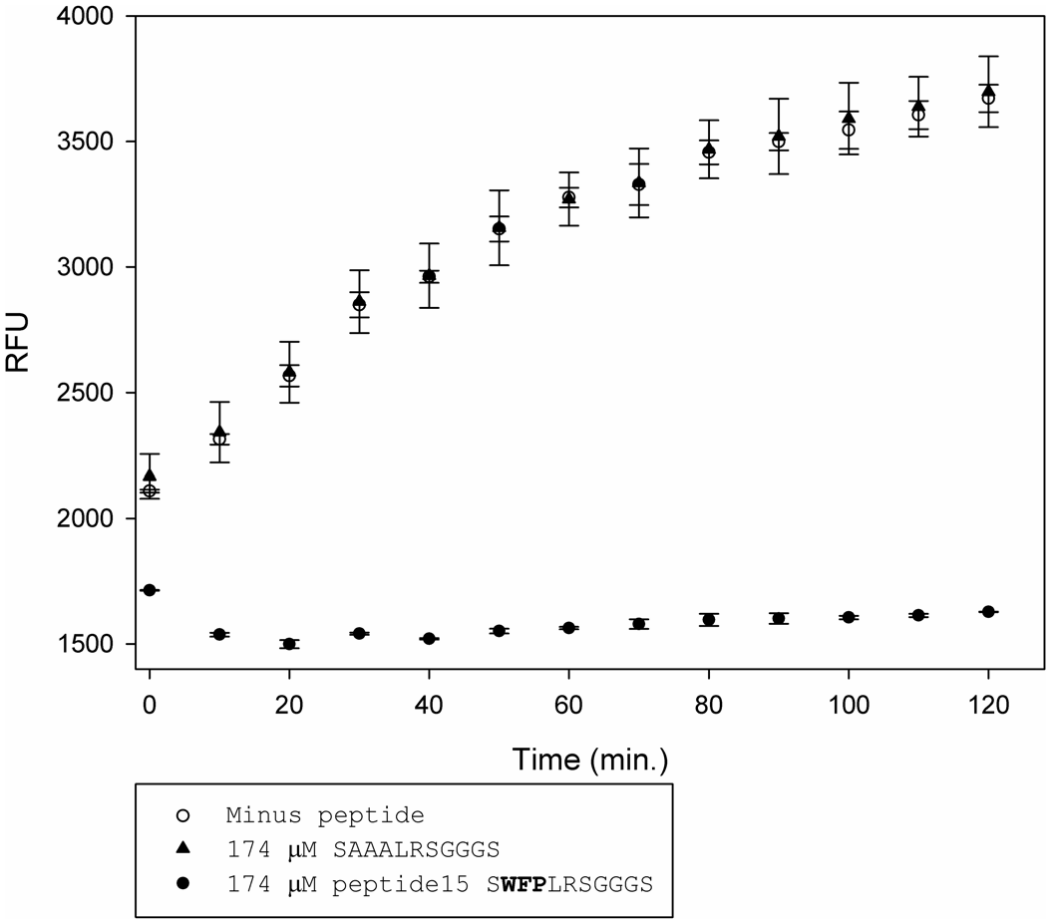
The WFP peptide motif is reponsible for Kly18 binding and inhibition. Pre-incubation of Kly18 with peptide15, (NH_2_)-SWFPLRSGGGS-(CONH_2_), and a control peptide (NH_2_)-SAAALRSGGGS-(CONH_2_), demonstrated that Kly18 inhibition could only be achieved with peptide15. Therefore, we concluded that the WFP motif was not only important for Kly18 binding but also for Kly18 inhibition. Error bars represent standard deviations between two experiments.

### Phage ELISA

Kly18 wells were coated and blocked as above. The following washes and incubations were performed at room temperature. After blocking, the plates were washed three times in PBS-T and one hundred µl solutions of the individual phage clones diluted 1∶1 in 4% BSA/PBS were added in a concentration of 1×10^10^ pfu/ml and further incubated for one hour. Wells were washed ten times with PBS-T and next 100 µl of peroxidase conjugated mouse anti-M13 monoclonal antibody diluted 1/1000 in 2% BSA/PBS, pH = 7.4 was added and incubated for one hour. The plates were washed ten times and detection was carried out by adding 100 µl of an OPD solution (1 OPD-tablet dissolved in 3 ml H_2_O and 5 µl 30% H_2_O_2_). The reaction was stopped by adding 100 µl of 1 M H_2_SO_4_ and absorbance values (*A*) were measured at 490 nm using an immunoreader (Emax).

**Table 1:** Peptides used in the truncation study.

Name	Sequence
15-0	(NH_2_)-SWFPLRSGGGS-(CONH_2_)
15-1	(NH_2_)-SWFPLRSGGG-(CONH_2_)
15-2	(NH_2_)-SWFPLRSGG-(CONH_2_)
15-3	(NH_2_)-SWFPLRSG-(CONH_2_)
15-4	(NH_2_)-SWFPLRS-(CONH_2_)
15-5	(NH_2_)-SWFPLR-(CONH_2_)
15-6	(NH_2_)-SWFPL-(CONH_2_)
15-7	(NH_2_)-SWFP-(CONH_2_)
15-8	(NH_2_)-WFP-(CONH_2_)
15-9*	(NH_2_)-WFPLRSGGGS-(CONH_2_)
15-10	(NH_2_)-WFPLRSGGG-(CONH_2_)
15-11	(NH_2_)-WFPLRSGG-(CONH_2_)
15-12	(NH_2_)-WFPLRSG-(CONH_2_)
15-13	(NH_2_)-WFPLRS-(CONH_2_)
15-14	(NH_2_)-WFPLR-(CONH_2_)
15-15	(NH_2_)-WFPL-(CONH_2_)
15-16	(NH_2_)-SWAPLRSGGGS-(CONH_2_)

Shown are all possible truncation combinations of peptide15, while still retaining the WFP motif. Each structure is given a number. The peptide15 lead structure has the number 15-0. Structure 15-9 was too hydrophobic for synthesis.

**Figure 5:**
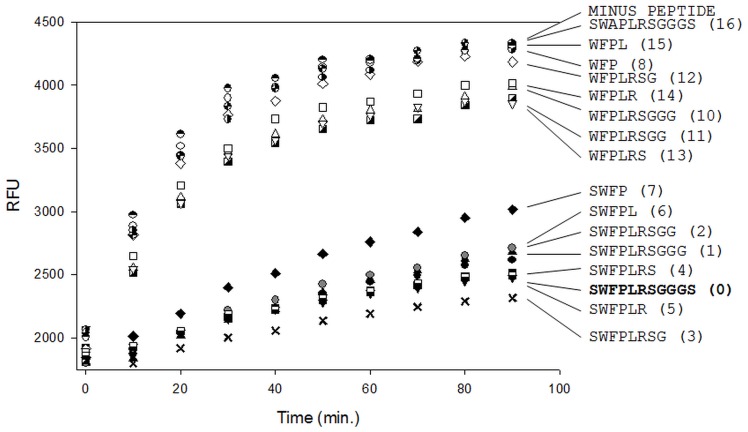
Screening of truncated peptides demonstrates that a tetra-peptide version of peptide15 can inhibit Kly18. Different length versions of peptide15 were tested for Kly18 inhibitory activity. All peptides were screened in a relatively high single concentration (87 µM) to reveal if omission of single amino acid residues displayed dramatic effects on Kly18 inhibition. The data demonstrated that peptide15 can be limited to (NH_2_)-SWFP-(CONH_2_) (structure 15-7) and still inhibit Kly18.

### Estimation of Peptide-phage Binding Affinity by the Use of Inhibition ELISA

Nunc Maxisorp plates were coated with Kly18 and blocked as described earlier. Titration curves of each clone revealed the phage concentration that gave half-maximum response, and this concentration was used for the individual inhibition assays. The inhibition assay was performed essentially as described previously [28]. Briefly, the individual phage clones were incubated with decreasing concentrations of Kly18 in 4% skimmed milk/PBS for 1 hour with extensive mixing. The mix was added to Kly18 coated and blocked wells and the unbound free phage were allowed to bind for 1 hour. Wells were washed with PBS-T five times and developed as above. The absorbance values were measured at 490 nm after 30 min of incubation at 22°C. Absorbance values determined at each Kly18 concentration (*A*) were divided by the absorbance measured in the presence of zero Kly18 (*A*_0_), thereby yielding normalized values (*A*/*A*_0_). These values were plotted against the Kly18 concentration to construct the inhibition curve. Curve fitting revealed the Kly18 concentration required for 50% inhibition (I_50_) as described previously [28]. The assay was performed in triplicate.

**Figure 6:**
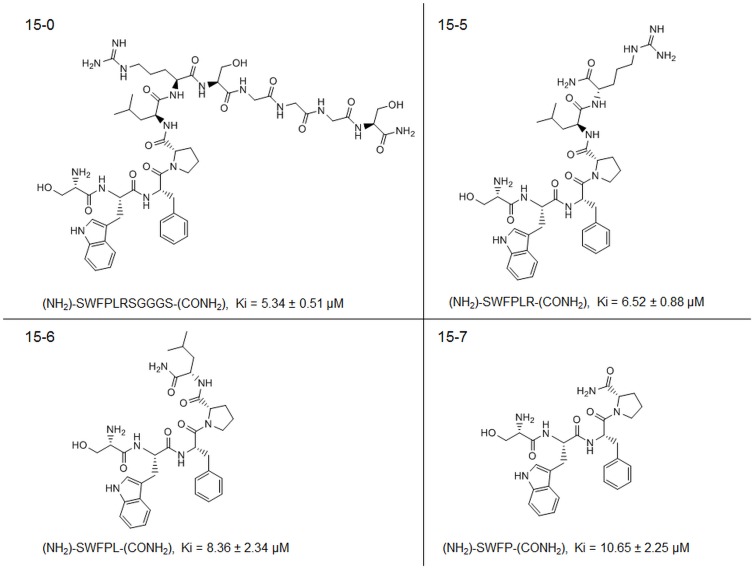
The tetrapeptide displays a Ki value similar to that of the peptide 15 lead structure. Shown are chemdraw structure models of the peptide versions 15-0, 15-5, 15-6 and 15-7. All truncated versions of peptide15 and intact peptide15 had low micromolar Ki values.

### Subcloning, Expression and Purification of the Maltose Binding Protein-peptide15 Fusion Protein

Peptide15 phage single stranded DNA was isolated as above and 25 ng of DNA was used as template for amplification of the peptide15 DNA by PCR using the M13KE insert extension primer and the −96 gIII sequencing primer. The programme used was 25 cycles of 95°C for 30 sec., 55°C for 30 sec. and 72°C for 30 sec. The PCR product was isolated as a single DNA band after agarose gel electrophoresis and digested with *Acc65I* and *EagI1*. The peptide15 DNA with flanking sequences was again purified after agarose gel electrophoresis. The peptide15 DNA was subcloned into *Acc65I/EagI* digested pMAL-pIII vector by T4-DNA ligase and transformed into chemocompetent TG1 cells. Clones were amplified, sequenced and preserved as glycerol stocks. A MBP-peptide15 clone was used for fusion-protein production by expansion in LB-medium (supplemented with 10 mM MgCl_2_, 0.2% glucose and 1 mM ampicillin) and when OD_600_ = 0.6 was reached the culture was induced with 1 mM IPTG for 3 hours at 30°C. The periplasmic fraction was isolated according to [29] and extensively dialysed into buffer A (20 mM Tris-HCl, 200 mM NaCl, 1 mM EDTA, pH 7.4). The dialysed fraction was applied to a 1 ml MBPTrap HP column at a flow rate of 1 ml/min and following extensive column wash with buffer A, MBP-peptide15 was eluted with 100% buffer B (20 mM Tris-HCl, 200 mM NaCl, 1 mM EDTA, 10 mM Maltose, pH 7.4). After dialysis into PBS, MBP-peptide15 purity was confirmed by SDS-PAGE.

**Figure 7:**
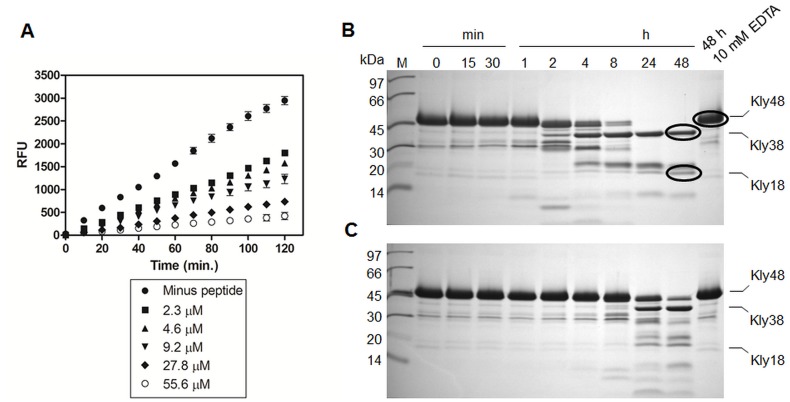
SWFPL inhibits the proteolytic activity of intact Karilysin (Kly48) and delays the auto-processing of Kly48 to Kly18. A) Kly48 was pre-incubated with SWFPL in varying concentrations (2.3 µM to 55.6 µM) followed by addition of the substrate FITC-casein. The SWFPL inhibitory effect was seen as a decrease in Relative Fluorescence Units (RFU) in a dose-response manner. Error bars represent standard deviations between three experiments. B+C) Intact Karilysin (Kly48) at 13 µM was incubated alone (B) or with the peptide inhibitor (C) at 455 µM. At indicated time points samples were withdrawn for SDS-PAGE analysis. It is clear that SWFPL inhibited the time-dependent autoprocessing of Kly48 to Kly38 and Kly18. Even after 48 hours a significant amount of Kly48 was still present.

### Kly18 Detection by MBP-peptide15 ELISA

Kly18 and human MMP-3 catalytic domains were coated at the concentration of 0.66 µM. Blank wells were included for background determination. All wells were blocked in 4% BSA/PBS for 1 hour. After wash with PBS-T (5 times), MBP-peptide15 in 2% BSA/PBS diluted to 25 µg/ml was incubated for one hour with shaking. After wash with PBS-T (5 times), a monoclonal anti-MBP HRP-conjugate was added diluted 1∶5000 in 2% BSA/PBS. Wells were washed ten times in PBS-T and developed with OPD substrate as above. To demonstrate that binding to Kly18 was mediated by peptide15 and not by MBP itself, control ELISA experiments were performed using MBP instead of MBP-peptide15.

**Figure 8:**
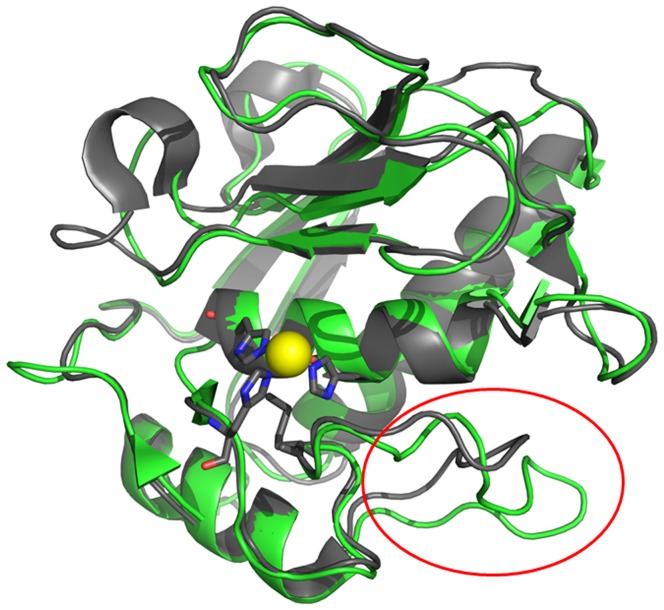
Structural alignment of the catalytic domains from Karilysin (Kly18) and human Matrix Metalloprotease-3 (MMP-3). MMP-3 is seen in green and Kly18 is seen in grey. The figure was prepared using Pymol (DeLano Scientific LLC) and the coordinate files 1CQR (MMP-3) and 2XS3 (Kly18). The structure alignment was performed using the ‘align’ function in PyMol. The Kly18 was structurally similar to MMP-3 as well as other mammalian MMP’s (MMP-1 to 3, MMP-7 to MMP-14, MMP-16 and MMP-20) (Cerda-Costa *et al.* 2011). The figure displays the zinc (yellow sphere) that is coordinated by the three active site histidines (H155, H159 and H165). The specificity loop is indicated with a red circle.

### Assay for Monitoring Peptide15 Inhibitory Activity towards Kly18

Kly18 and Kly48 protease activities were monitored essentially as described [6]. One hundred µl working volumes were used in black untreated polypropylene microtitre plates and FITC-casein was used as the substrate. Assays were performed at 37°C using 500 nM of Kly18 (or Kly48) in assay buffer (100 mM Tris-HCl, 5mM CaCl_2_, pH 8.0), at a FITC-casein concentration of 25 µg/ml. Released fluorescence was measured using a micro-titer plate reader at excitation/emission wavelengths of 485/538 nm. Peptides were dissolved in Milli Q water and added in varying molarities. The assay setup was as follows; Kly18 (or Kly48) was diluted in assay buffer together with peptide, followed by 30 minutes incubation on a vertical shaker at 22°C. Then FITC-casein was added and fluorescence formation was monitored at 37°C for 90 minutes with measurements every ten minutes. For estimation of inhibition constants (Ki) for peptide inhibition of Kly18, a fixed amount of Kly18 (500 nM as above) was incubated with varying concentrations of FITC-casein (5–25 µg/ml) in the presence of varying amounts of Kly18 peptide inhibitor (0–10 µM). Enzyme velocities were plotted, curves fitted and determination of Ki was performed using GraphPad Prism 5.04 using the macro for competitive inhibitor.

**Figure 9:**
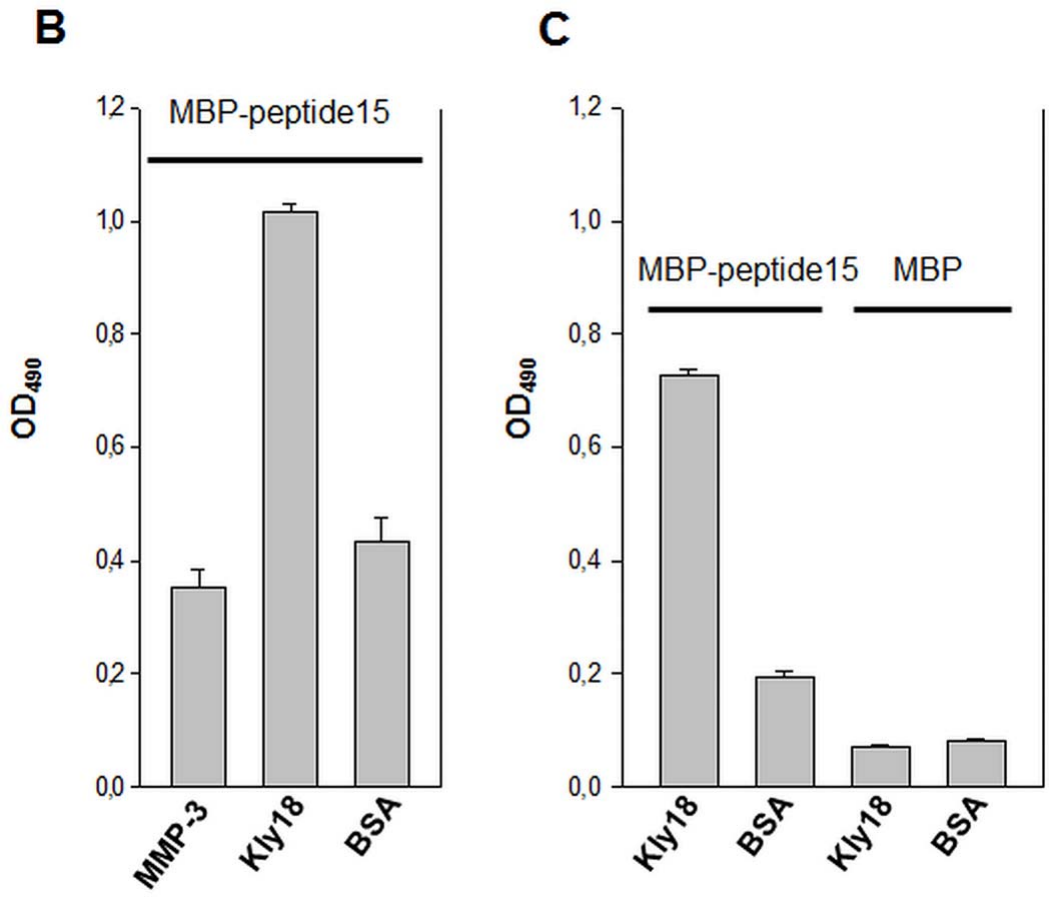
Peptide15-MBP is specific for Kly18. A) The peptide15-MBP fusion protein interacted exclusively with Kly18, thereby demonstrating that peptide15 was specific for Kly18. **B)** To demonstrate that binding to Kly18 was mediated by peptide15 and not by MBP itself, control experiments were performed using MBP instead of MBP-peptide15. Error bars represent standard deviations between three experiments.

### Assay for Monitoring the Effect of Peptide15 on the Auto-processing of Kly48

Karilysin (Kly48) at 13 µM was incubated at 37°C in 50 mM Tris-HCl, 2.5 mM CaCl_2_, 0.02% NaN_3_ pH 8.0 alone or in the presence of the peptide SWFPL at 455 µM. At different time points samples were withdrawn and the Kly48 autocatalytic processing was monitored by SDS-PAGE using 10% gels and the Tris-HCl/Tricine buffer system [30]. Gels were stained with 0.1% Coomassie Brilliant Blue R-250 in 10% acetic acid followed by destaining. Samples were run alongside a Low Molecular Weight SDS-PAGE Marker.

## Results and Discussion

### Isolation of Kly18 Binding Peptide-phage

Peptide phage display is an excellent tool for fast identification of lead structures that can be further modified as protease inhibitors [31]. In the present study 7-mer peptide libraries were used for bio-panning against the catalytic domain from Karilysin (Kly18). A cyclic 7-mer disulfide-constrained library (ACX_7_CGGGS) and a linear 7-mer library (X_7_GGGS) were mixed and used for selection on Kly18. Both libraries were fused to the N-terminal of protein III and displayed on phage. After four bio-panning rounds against surface-immobilized Kly18 a large enrichment in Kly18 binding phage was observed (>500 compared to the BSA control, data not shown). Nineteen clones were amplified and phage ELISA revealed seven Kly18-binders, peptide-phage 9,11,13,14,15,16,19 (Figure 1A). All seven peptide-phage displayed apparent affinities in the low micromolar range (Figure 1B). Only linear binders were isolated suggesting that the linear binders were of higher affinity than any potential cyclic peptides present in the library. In bio-panning experiments against other protein targets, we have used the same approach of mixing linear and cyclic libraries, but in these cases we isolated constrained peptides as well as linear peptides (unpublished). This suggested that we could exclude the possibility of a higher growth rate of the linear peptide library as the reason why we only isolated linear binders. All identified Kly18-binding peptide-phage (except peptide13) had the WXP motif, clearly demonstrating that these amino acids are important for Kly18 binding (Figure 1B). Interestingly, no peptides were found with the WXP motif in position 1–3 and all the peptides had the WXP peptide motif placed in position 2–4, thereby suggesting that an amino acid is essential in position one prior to the WXP motif for structural stability. Furthermore, it was evident that the X-position in the Kly18-binding motif was dependent upon basic amino acids (arginine/lysine) or bulky hydrophobic residues (leucine/phenylalanine) (Figure 1B). A decision was made to further focus on peptide15 (SWFPLRSGGGS).

### Free Soluble Peptide15 Inhibits Kly18 Proteolytic Activity

Peptide15 was chemically synthesised for activity testing. Initially, the peptide was synthesised in its entirety, mimicking all of the sequence displayed on phage. The peptide15 N-terminal was free during panning, however the C-terminal was fused to the phage. Consequently, peptide15 did not have a free negatively charged carboxylate at its C-terminal during panning. Therefore, in order to limit an effect of a C-terminal carboxylate, peptide15 was synthesised with a C-terminal amidation to block any unwanted C-terminal effects. The peptide15 synthesised was therefore (NH_2_)-SWFPLRSGGGS-(CONH_2_). The Kly18 proteolytic activity was monitored with FITC-casein as the substrate and peptide15 inhibited Kly18-mediated FITC-casein cleavage in a dose-response manner (Figure 2). Furthermore, peptide15 displayed the characteristics of a competitive inhibitor as shown from a 1/V-1/S plot of peptide15 inhibition of Kly18. As seen from the Lineweaver-Burke plot the Y-intercept is approximately the same for un-inhibited Kly18 as Kly18 inhibited with 10 µM peptide15 and 30 µM peptide15, respectively (Figure 3). This suggested that peptide15 was a competitive inhibitor of Kly18.

To investigate the importance of the peptide15 WFP motif for Kly18 inhibition a control peptide was chemically synthesised with the WFP motif replaced by a triple alanine, (NH_2_)-SAAALRSGGGS-(CONH_2_). Even in a very high dose (174 µM) the control peptide had a curve similar to that of Kly18 with no peptide present, thereby demonstrating that the WFP motif was crucial for inhibition of Kly18 (Figure 4).

### Peptide15 Truncation Study and Comparative Kinetic Analysis

Peptide15 was synthesised in its entirety, including the linker sequence attached to the phage (GGGS) and the question arose if the amino acids flanking the WFP motif were necessary for Kly18 inhibition. To answer this, fifteen truncation variants of peptide15 were designed, termed 15-1 to 15–16 and the peptide15 lead structure was named 15-0 (Table 1). Peptide version 15-9 (WFPLRSGGGS) was too hydrophobic for synthesis but all others could be produced. Initially all peptide versions were screened in a relatively high single concentration (87 µM) to reveal if omission of single amino acid residues displayed dramatic effects on Kly18 inhibition. The peptide15 truncated version (NH_2_)-SWFP-(CONH_2_) retained efficient inhibitory potency but the peptide (NH_2_)-WFP-(CONH_2_) was completely inactive towards Kly18 (Figure 5). The data clearly demonstrated that the serine in position 1 was crucial for inhibition of Kly18 as all peptide versions lacking this serine (15-8,15-10,15-11,15-12,15-13,15-14,15-15) lost most or all of their Kly18 inhibition capacity (Figure 5). Therefore, the Kly18 inhibiting peptide motif was expanded to (NH_2_)-SWFP-(CONH_2_). A possible explanation is that in absence of serine the presence of a charged amino group attached directly to the tryptophan of the (NH_2_)-WFP-(CONH_2_) motif could interfere with the binding and inhibitory mechanism of the peptide. Interestingly, full length peptide15 with an alanine substitution in position 3 (15-16), (NH_2_)-SWAPLRSGGGS-(CONH_2_), was also inactive towards Kly18 (Figure 5). This demonstrated that the phenylanaline was also crucial for the inhibitory activity of peptide15.

Detailed kinetic analysis of peptides 15-0.,15-5., 15-6. and 15-7. revealed similar Ki values in the low micromolar area (Figure 6). This demonstrated that peptide15 can be truncated to the tetra-peptide (NH_2_)-SWFP-(CONH_2_) without compromising inhibitory potency.

### Peptide15 (SWFPL) Inhibits the Proteolytic Activity of Intact Karylysin (Kly48) and Delays the Auto-processing of Kly48 to Kly18

We next wanted to test if the inhibitory effect of peptide15 on Kly18 could be reproduced on intact Karilysin (Kly48). The experiment was performed essentially as described for Kly18 and the results demonstrated a clear dose-response inhibition of Kly48 by (NH_2_)-SWFPL-(CONH_2_) (Figure 7A). Kly48 is unstable and auto-processes itself into Kly38 and Kly18 over time. Consequently, it was important to establish if SWFPL could modulate the Kly48 auto-processing. As seen from the SDS-PAGE gels in Figure 7B and 7C, (NH_2_)-SWFPL-(CONH_2_) inhibited the auto-processesing efficiently and even after 48 hours there was still a considerable amount of Kly48 present. Taken together these two studies confirmed that peptide15 inhibited the proteolytic activity of Kly48 and this dataset strongly suggested that it may be possible to use peptide15 to lock Karilysin in the intact Kly48 form, and use that complex for crystallisation trials. This could reveal the structure of the intact enzyme.

### Expression of MBP-peptide15 Fusion Construct and Specificity Test

In figure 8 a structural alignment of Kly18 and the catalytic domain of human MMP-3 is shown and as is the case with most human MMP’s (MMP-1, MMP-7-14), Kly18 and human MMP-3 are structurally similar [22]. It was therefore important to establish if peptide15 was a general binder of MMP catalytic domains. To develop a probe for cross-reactivity tests, peptide15 was expressed as a MBP N-terminal fusion protein and purified in a simple one-step purification strategy. Purity was judged to be greater than 90% by SDS-PAGE (data not shown) and the MBP-peptide15 was used in ELISA to test cross-reactivity towards human MMP-3. MBP-peptide15 bound Kly18 but not MMP-3, thereby underlining peptide15 specificity (figure 9A). By performing a control ELISA experiment using MBP instead of MBP-peptide15, we could confirm that the binding observed was due to the specific interaction between peptide15 and Kly18 (Figure 9B).

As MMP catalytic domains are highly similar it is a challenge to find specific peptides. This was demonstrated in a recent study, which identified a cyclic MMP-9-binding peptide (CTTHWGFTLC) by phage display with high affinity. However, this peptide also had high affinity for MMP-2 [32]. Peptide phage display has also been used to identify substrate specificity of individual MMPs and by using a 15-mer peptide library Deng and co-workers demonstrated that collagenase 3/MMP-13 has a preference towards the peptide sequence GPLGMRGL. But three other MMPs (stromelysin-1/MMP-3, gelatinase B/MMP-9 and collagenase 1/MMP-1) had lower activity towards the isolated peptide substrate [33]. Furthermore, a recent study demonstrated that a unique MMP-11 substrate peptide could be isolated by phage display, which explained why MMP-11 does not have any activity towards substrates specific for other MMPs [34]. Consequently, one could argue that the peptide15 mode of action is that of a substrate competitive inhibitor. This is especially relevant due to the fact that the exact peptide sequence needed for Kly18 substrate cleavage is yet to be fully elucidated [6]. To explore this possibility peptide15 was incubated with or without a large excess of Kly18 and the mixture was analyzed by liquid chromatography mass spectrometry. The results demonstrated that peptide15 could not be cleaved by Kly18 as the mass peak m/z = 1149, corresponding to (NH_2_)-SWFPLRSGGGS-(CONH_2_), was found in both preparations and no cleavage products were found (data not shown).

The development of MMP inhibitors is highly relevant, due to the involvement of MMPs in several pathologies [23]. Some candidate molecules act by blocking the MMP-active sites via binding to the catalytic Zn^2+^ through chelators such as hydroxamate [23]. These approaches have been successful for MMP inhibition. However, the inhibitors have a broad specificity towards other MMPs due to very similar active site geometries of matrix metalloproteinases [23]. An alternative approach to specific MMP inhibitor design is to utilize larger molecules, such as peptides, that interact with the active site as well as exosites unique to individual MMPs [35]. Kly18 contains the metzincin MMP 3-histidine zinc-binding motif (HEXXHXXGXXH) and Kly18 was previously inhibited by small molecules chelators (EDTA, 1,10-phenanthroline) similar to mammalian MMPs [6]. Furthermore, the active site of the Kly18 structure had the classical MMP composition with small deviations in the specificity loop (seen as a red circle in Figure 8) [22]. As peptide15 binds exclusively to Kly18 and at the same time inhibits the enzyme, peptide15 may act by a combined effect of active site targeting and specificity loop exosite interaction. However, detailed structural analysis of Kly18-peptide15 co-crystals will provide final proof on the inhibition mechanism.

### Conclusions

Periodontitis is a devastating disease of great discomfort to affected patients. Furthermore, recent findings link periodontitis to inflammatory diseases like rheumatoid arthritis, thereby further underlining the importance of disease treatment and containment [36]. Inhibitors of the periodontopathogen-derived proteases by small molecules, peptides or antibodies represent a rational approach to disease management. Inhibitors toward the most relevant proteases, of which Karilysin could be one, might be applied as a combination therapy in the fight against the periodontopathogens of the ‘red complex’. These inhibitors could be formulated in existing media, such as mouth wash, tooth paste or even chewing gum.

In this study, the first inhibitor of Karilysin was identified and characterized. The results supplied us with a detailed understanding of which amino acid residues were important for the inhibitory function of peptide15. The peptide will be a valuable probe in structure-function studies of Karilysin and detailed co-crystallisation studies can reveal the underlying molecular mechanism for the inhibition. From co-crystallisation structural data it may be possible to design peptidomimetics with improved Ki-values.
